# Cronkhite- Canada syndrome; a case report and review of the literature 

**Published:** 2016

**Authors:** Mohammad Taghi Safari, Shabnam Shahrokh, Shahram Ebadi, Amir Sadeghi

**Affiliations:** *Gastroenterology and Liver Diseases Research Center, Research Institute for Gastroenterology and Liver Diseases, Shahid Beheshti University of Medical Sciences, Tehran, Iran*

**Keywords:** Cronkhite- Canada syndrome, Hamartomatous polyp, Diarrhea

## Abstract

Cronkhite- Canada syndrome (CCS) considered as a rare and non-hereditary disorder. Gastrointestinal polyposis and diarrhea along with some extra signs and symptoms such as hypoproteinemia, and epidermal manifestations are recognized in this syndrome. The pathophysiology of this syndrome is not completely understood and it seems that inflammatory processes may be involved. We present a 50 year-old man with hamartomatous polyps throughout the colon and long-lasting diarrhea not responding to typical therapies during three years.

## Introduction

 Cronkhite-Canada syndrome (CCS) is a rare, non-hereditary condition which characterized by gastrointestinal polyposis associated with diarrhea, hypoproteinemia, and epidermal manifestations such as cutaneous hyperpigmentation, alopecia, onychodystrophy, and atrophic nail change (1). The overall mortality rate has been reported to be approximately 60%, and the mean age of presentation is 59, however, the majority of cases identified in age older than 50 (2). The other GI complications, which are present in this disorder, are protein-losing enteropathy, malnutrition, infection, and gastrointestinal bleeding. The pathogenesis of Cronkhite-Canada syndrome is not fully identified and it is still unknown. Despite the rare correlation of this syndrome with malignancies in previous reports (3), recent studies have demonstrated the association of CCS with gastrointestinal cancers, including gastric cancer and colorectal cancer via development of adenomas and/or carcinomas polyps (4-9). From the endoscopic and histologic view, they are similar to other polyposis syndromes, including familial adenomatous polyposis (10), Peutz-Jeghers syndrome, Cowden disease, hyperplastic polyposis and juvenile polyposis (11, 12). Among them, juvenile polyps have the most histologic similarity with CCS polyps despite they have an extensive sessile base. Thus, determination of CCS would be according to other criteria and not only based on polyp histology. In this study, we describe a 50- year-old Iranian male with Cronkite- Canada syndrome presenting with severe chronic diarrhea, alopecia, skin hyperpigmentation and onychodystrophia of the fingers. 

## Case Report

**Figure 1:**
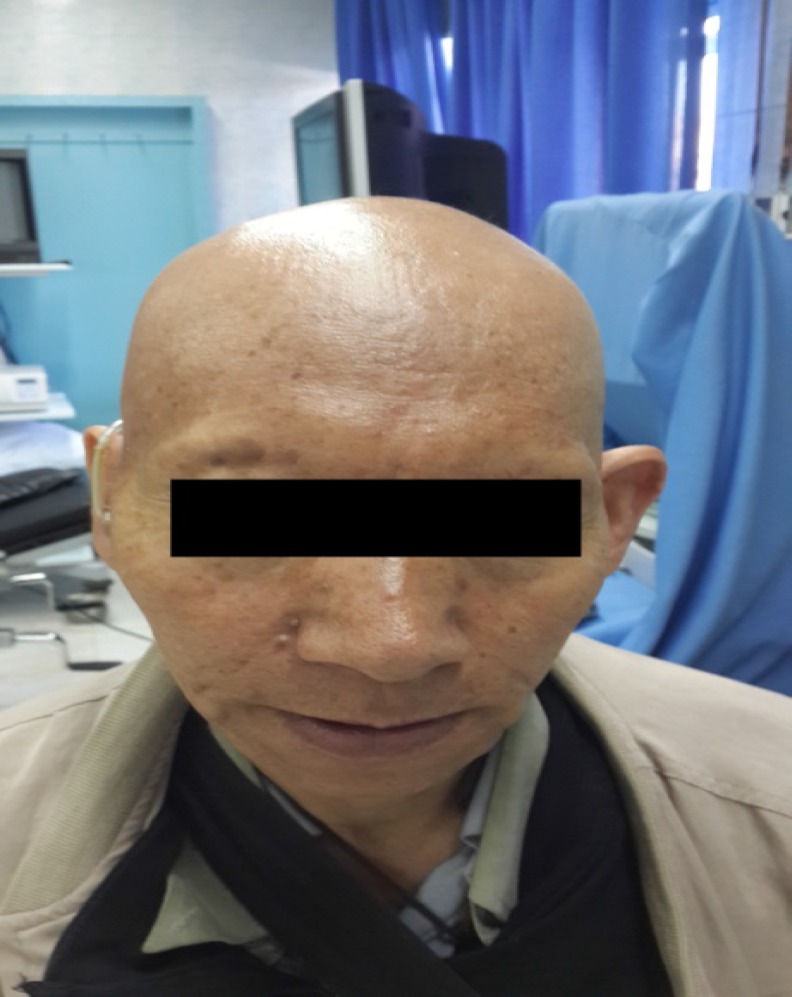
Alopecia and hair loss identified in patient with CCS

**Figure 2:**
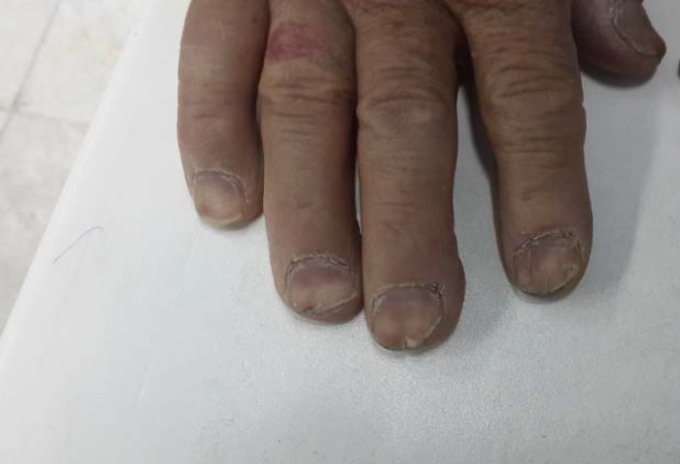
Hyperpigmentation of the hands and fingers are present in this case

**Figure 3:**
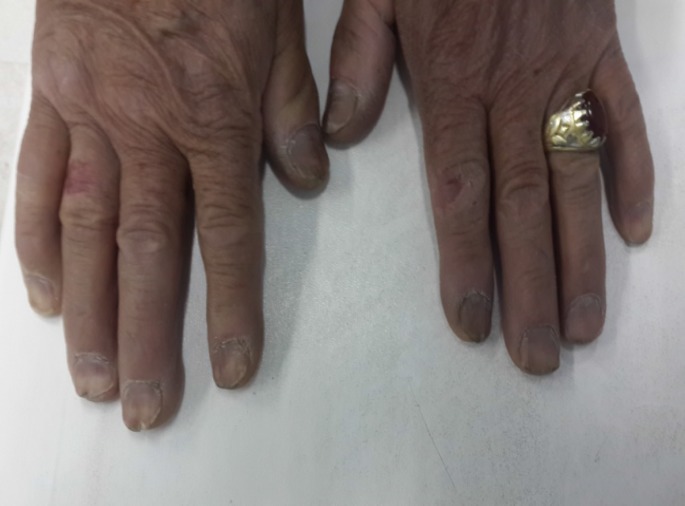
Onychodystrophy and atrophic nail change in patient with CCS

A 50-year-old Iranian male referred to our clinic with complaint of chronic diarrhea, which lasted since three years ago. The diarrhea wasn’t related to food consumption and had not even obviated by previous fasten diets. This chronic watery diarrhea was not accompanied with any blood, mucosa, fat or oil and happened at least ten times a day and sometimes occurred at night and did not obviate by colestiramina, loperamide, bismuth or other frequent antibiotic medications. Furthermore, the patient had fatigue, but denied any fever, abdominal pain, nausea or vomiting during. The patient did not have any history of celiac disease, cancers or polyposis syndromes in his family. The physical examination of the patient revealed alopecia and hair loss which occurred on the scalp, eyebrows, eyelashes, axilla, and limbs (Figure 1), as well as hyperpigmentation of the hands and feet (Figure 2) accompany with onychodystrophia of the fingernails (Figure 3), which had been started three years before the admission, simultaneously with severe diarrhea. 

Abdominal examination was normal and the rest of the physical examination was not promising. Laboratory test revealed that his total count of white blood cell (WBC) was 4900/μL (normal 4-10×10^3^/μl), red blood cell (RBC) was 5.48×10^6^/μL (4.5-5.8×10^6^/μL), platelet was 295×10^6^ /μL (150-450×10^6^), and Hb was 16.1 gr/dl (normal 14-17 gr/dl). C-reactive protein (CRP) was negative. According to the result, hemoglobin, glucose, platelets, urea, transaminases, alcaline phosphatase, creatinine, bilirubin, triglycerides, cholesterol, folic acid, and ferritin were all in a normal range (table 1). 

The patient underwent Gastroscopy and esophagus, gastric fundus and body were normal. Colonoscopy was performed and one hamartomatous polyp was detected at rectum. During the procedure the biopsy was taken and the serological tests, including anti-tissue transglutaminase (tTG) antibodies and Anti EMA-IgA were done for possible presentation of celiac in this case. The immunoglobulin A (IgA) was 197 Mg/dl, which was also in the normal range (70-400 Mg/dl).

**Table 1:** Complement tests did not indicate any remarkable findings

**Parameter**	**Result**	**Unit**	**Normal Range**
WBC	4.9	X10^3^/μl	4-10
RBC	5.48	X10^6^/μl	4.5 -5.8
HGB	16.1	gr/dl	14-17
HCT	48.1	%	42-50
MCV	87.8	fl	80-100
MCH	29.4	pg	27-33
MCHC	33.5	gr/dl	31-36
PLT	295	X10^6^/μl	150-450
ECR	6	MM	M>50: 0-20
TSH	1.2	Mlu/l	0.3-4
T3	168.0	Ng/dl	800-200
T4	8.3	Ug/dl	4.5-12.5
ALP	148	lu/l	44-147
CREATINE	0.8	Mg/dl	0.7-1.3
AST	34	Lu/L	10 to 40
ALT	38	lu/l	7-56
IgA	197	Mg/dl	70-400
Anti-tTG (IgA)	Neg	Mg/dl	<20
Anti-EMA IgA	Neg	u/l	1:10

Based on alopecia and hair loss, hyperpigmentation of the hands and fingers with onychodystrophia of the fingernails, severe diarrhea, and detection of hamartomatous polyp in rectum the case was considered as Cronkhite Canada syndrome. Patient treated by administration of antibiotics and anti-inflammatory medication with 20mg of prednisolone a day, which tapered after 4 weeks to 5 mg once which resulted in significant rapid improvement in reduction of the severity of the watery diarrhea. 

## Discussion

Despite some reports addressing the association of CCS with enhanced antinuclear antibody (ANA) and immunoglobulin IgG4 levels in CCS polyps, the etiology of CCS is unknown (11, 13). Although endocrine system deficiency and infection such as Helicobacter pylori also noted as potential factors contributed to the CCS etiology in some studies (14-16). Diagnosis of CCS is usually based on history, physical examination, as well as histology and endoscopy findings. It has been also reported that CCS harbor a smooth male predominance with (3:2, male to female)(17). Frequent GI-related symptoms were identified in patients with CCS including diarrhea, abdominal pain, weight loss, nausea, anorexia, haematochaezia, vomiting and hypo-/dysgeusia (18, 19). The previous epidemiological study indicated the hypogeusia as the most common features in 40.9% of the cases followed by diarrhea (35.4%),, abdominal discomfort (9.1%), and alopecia in 8.2% patients (19). Several treatments have been indicated for CCS patients, such as nutritional diet, immune suppression, administration of antibiotics, as well as surgery, azathioprine, and glucocorticoids, anabolic steroids (20-25). However, due to the rarity of the entity, there hasn’t been the standard gold and specific medication for index patient and this leads to a poorer outcome. In our study, the patient treated by administration of antibiotics beside anti-inflammatory medication with 5mg prednisolone which leads to a significant improvement in reduction of watery diarrhea severity. There have been several studies about the efficiency of prednisolone administration and clinical improvement of CCs cases (26-28). It has been also demonstrated that approximately 9–15% of the cases attributed to malignant conditions (9, 11, 21). It has been reported that the long-term prognosis for CCS patients is rather poor. In their study on 55 cases with CCS, Daniel et al. revealed a 55% mortality rate for those patients (8). In the present study, we describe a 50-year-old Iranian patient with Cronkhite-Canada syndrome presenting with sever chronic diarrhea, alopecia, hyperpigmentation of the hands and fingers and onychodystrophia. In line of our study, Hee Yun, reported a case of CCS associated with several hamartomatous polyps from the stomach to the colon. The colon polyps were revealed to be adenocarcinoma in situ and serrated adenoma (28). Consistent to our findings, in another paper by Chakrabarti, a 68-year-old male presented with weight loss and no history of fever, abdominal pain or any sign of blood in watery diarrhea. Although, they revealed a weight loss which we didn't observe in our case (29). Adenomatous polyps are one of the most frequent lesions present in CCS patients that are also the precursors of colorectal cancer (30-33). In this regard, Christopher M, reported a 70-year-old Caucasian male with CCS presenting severe adenomatous change within innumerable hamartomatous colonic polyps (32). Another study by Taro Isobeetal, reported a case of Cronkhite-Canada Syndrome associated with several gastric adenocarcinoma and multiple colon adenomas polyps (tubular adenoma) in the large intestine (31). Despite we didn’t detect any suspicious lesions in the stomach during the upper endoscopy, we observed one hamartomatous polyp in the rectum of the case. Seth Sweetser reported a case of Cronkhite–Canada syndrome presenting with adenomatous and inflammatory colon polyps. In contrast to our study, the case experienced a 22.68 kg (50 lb) weight loss, due to the appetite decrease (33). In another valuable report, C. Sellal et al. detected several non-hardened sessile polyps in the large intestine without malignancy potential, associated with gastric polyposis (34).

With the high morbidity and mortality of Cronkhite-Canada syndrome concomitant with the enigmatic etiology of this syndrome, it is highly recommended that implication of molecular study might be the best and only possible approach to unravel the mechanism underlying this phenotype.
